# Psychiatric Sequelae of Guillain-Barré Syndrome: Towards a Multidisciplinary Team Approach

**DOI:** 10.7759/cureus.7051

**Published:** 2020-02-19

**Authors:** Christopher Hillyar, Anjan Nibber

**Affiliations:** 1 Medicine and Dentistry, Barts Health NHS Trust, London, GBR; 2 Neurology, Oxford University Medical School, Oxford University Hospitals NHS Foundation Trust, Oxford, GBR

**Keywords:** guillain-barré syndrome, psychiatry, stress, anxiety, depression, fatigue, delirium, psychoeducation, multidisciplinary team, icu

## Abstract

Guillain-Barré syndrome (GBS) is a post-infectious autoimmune polyneuropathy. Recent research has highlighted that GBS is associated with the onset of psychiatric symptoms which represent a burden for patients and close relatives. However, acute psychiatric sequelae due to GBS may be misinterpreted as ‘intensive care unit (ICU) delirium’. This review outlines the existing evidence for the psychiatric symptoms associated with GBS with a view to improving psychoeducation of patients. The main psychiatric symptoms of GBS that have been reported in the literature include, stress, anxiety, depression, fatigue, sleep abnormalities, visual hallucinations, paranoid delusions, disorientation, terror and psychosis. These psychiatric symptoms, which occur during the acute phase of GBS, if not recognised and treated, may progress to long-term psychiatric problems that interfere with improvement of physical symptoms. A multidisciplinary team approach to the management of GBS may improve both physical and psychiatric recovery.

## Introduction and background

Guillain-Barré syndrome (GBS) is a post-infectious autoimmune polyneuropathy with an annual incidence of 1.5-1.7 per 100,000 [1-3]. The acute polyneuropathy manifests as bilateral symmetrical muscle paresis/paralysis (affecting balance, posture, joint mobility and gait), hyporeflexia/areflexia (usually affecting the calcaneal reflex), and sensory dysfunction (tingling and burning sensations) progressively ascending from the lower limbs. Occasionally, cranial nerve involvement causes unilateral facial palsy or facial diplegia [4]. The pathophysiology of GBS involves immune-mediated demyelination and/or axonal damage of peripheral nerves due to molecular mimicry between microbial antigens (e.g., Campylobacter jejuni, Mycoplasma pneumonia, cytomegalovirus, Epstein-Barr virus, influenza virus) and neuronal antigens. The subtypes of GBS have different geographical distributions, with demyelinating subtype predominating in Europe and USA and axonal subtype found more commonly in Asia, North Africa and South America [5].

The natural history of the disease involves an acute phase characterised by physical disability which reaches a plateau within two weeks. Timely clinical management is required due to the risk for respiratory failure resulting from dysfunction of the phrenic nerve, with 20%-30% of patients requiring assisted ventilation [1, 6, 7]. Early signs of respiratory failure include tachypnoea, tachycardia and air hunger, while late signs include use of accessory respiratory muscles, paradoxical breathing and orthopnoea due to diaphragmatic paresis [7]. After the acute phase, patients enter a rehabilitation phase with most returning to usual daily activities within three years, with greater improvements amongst younger patients. However, at least 20% of GBS patients experience some long-term disability [1, 8-10].

Although GBS is treatable with plasmapheresis and/or intravenous (IV) immunoglobulin (Ig), many patients experience residual psychiatric symptoms that lead to physical disability, behavioural dysfunction and pain [11]. Despite the heavy burden that psychiatric symptoms place on patients and their families, the psychiatric sequalae associated with GBS often go underappreciated due, in part, to the lack of recognition of their consequences. This review aimed 1) to outline the psychiatric sequalae of GBS and their effects on patients and close relatives, and 2) to review strategies for improving the management of GBS through a multidisciplinary team approach for the management of psychiatric symptoms.

## Review

A review of the recent literature suggests that GBS patients, and close relatives of GBS patients, are indeed at increased risk of psychiatric symptoms. Table 1 includes six case studies reporting anxiety, lethargy and sleep disturbance at the onset of physical disability, as well as residual psychiatric symptoms at one to four weeks after GBS onset [12-15]. Two case studies reported that psychiatric symptoms preceded onset of physical disability, including stress, depression, anxiety and amnesia [16, 17]. Table 2 includes 24 studies, including a total of 6,984 patients, that collectively reported that GBS causes psychiatric symptoms, including stress, anxiety, depression, fatigue, sleep abnormalities, visual hallucinations, paranoid delusions, disorientation, terror, and psychosis [11, 17-36]. Two studies concluded that close relatives of GBS patients also experience problems of daily living and social dysfunction and have psychological needs requiring consideration and support [23, 37]. As a result, GBS patients and relatives of GBS patients may benefit from psychosocial education to promote awareness of their psychiatric needs.

**Table 1:** Summary of case reports on psychological sequelae associated with Guillain-Barré syndrome (GBS) Adapted from [16].

Reference	Subject (age/sex)	Conclusion
Chemtob and Herriott [13]	24/F	Anxiety, lethargy, and sleep disturbance after the onset of physical disability
Neroutsos et al. [14]	20/F	Residual anxiety and depression during recovery phase
Brousseau et al. [12]	56/F	Depression, affective liability, anxiety, and agitation one week after onset of physical disability
	64/F	Depression and anxiety two weeks after onset of physical disability
	66/F	Depression and anxiety four weeks after onset of physical disability
Sangroula et al. [16]	24/M	Prior depression, anxiety, and amnesia associated with development of GBS
Tagami et al. [15]	65/M	Depression, anxiety, and fatigue three weeks after onset of physical disability
Weiss and Luke [17]	44/M	Prior stressful event associated with development of GBS

**Table 2:** Summary of research studies on psychological sequelae associated with Guillain-Barré syndrome (GBS) Adapted from [16].

Reference	Study characteristics	No. of GBS patients	Conclusions
Bahnasy et al. [10]	Prospective study	20	Residual psychiatric symptoms and sleep abnormalities associated with GBS
Bernsen et al. [37]	Retrospective study	86	Problems of daily living and social dysfunction affect relatives of GBS patients
Bernsen et al. [25]	Randomised, double-blind, placebo-controlled study	85	Residual depression associated with GBS; psychosocial health impaired at one year
Bussmann et al. [29]	Case series	20	Changes in residual fatigue independent of actual physical fitness of GBS patients
Cochen et al. [36]	Prospective study	139	Visual hallucinations and paranoid delusions peaked on day 9 in intensive care unit (ICU)-admitted GBS patients
Davidson et al. [22]	Prospective survey	884	Increased anxiety, depression, and fatigue in GBS patients
Eisendraht et al. [23]	Prospective study	8	Physical disability in GBS leads to anxiety, disorientation, and terror; educate patient and family with regards to psychological needs
Gao et al. [35]	Case series	49	Reduced sleep quality related to anxiety in GBS
Garssen et al. [38]	Case series	20	Physical exercise reduces anxiety in GBS independent of increased physical fitness of patients
Garssen et al. [30]	Case series	16	Residual fatigue in GBS is independent of nerve dysfunction measured using conventional nerve conduction studies
Garssen et al. [31]	Prospective study	100	Residual fatigue is independent of physical disability in GBS patients
Garssen et al. [24]	Randomised, double-blind, placebo-controlled study	80	Residual anxiety and depression associated with GBS is refractory to amantadine
Karkare et al. [27]	Prospective study	60	Sleep disturbance in GBS is associated with depression
Khan et al. [18]	Prospective study	76	Residual depression, anxiety, and stress associated with GBS
Kogos Jr. et al. [26]	Retrospective study	18	Residual depression is related to pain associated with GBS
Kuitwaard et al. [32]	Retrospective study	240	Severe residual fatigue experienced by 70% of GBS patients
Le Guennec et al. [19]	Retrospective study	13	Post-traumatic stress syndrome associated with GBS independent of anxiety and depression
Merkies et al. [33]	Retrospective study	113	Residual fatigue persists for years after recovery phase of GBS
Rhanjani et al. [28]	Retrospective study	90	Residual fatigue persists beyond recovery phase of GBS despite improvement in motor fatigue
Rekand et al. [34]	Retrospective study	50	Fatigue is a common symptom in GBS patients
Sharshar et al. [21]	Retrospective study	110	Anxiety should be evaluated early in order to provide psychological support to GBS patients
Tzeng et al. [11]	Retrospective study	4,548	High risk for development of psychiatric disorders in GBS patients necessitates regular follow-up
Weiss et al. [20]	Prospective study	49	Residual anxiety, acute stress disorder, depression, and psychosis associated with GBS
Witsch et al. [39]	Retrospective study	110	Residual fatigue, chronic pain, and low energy levels associated with GBS

A large study by Tzeng et al. of 4,548 GBS patients demonstrated significantly increased cumulative risk of neuropsychiatric disorders in comparison to 13,544 matched controls (10.35% vs 7.5%; hazard ratio: 4.281, P < 0.001) [11]. Assisted ventilation, respiratory failure, pulmonary complications, cardiac complications, and systemic infection were associated with increased risk for developing psychological disorders. However, medical care in a local hospital as opposed to a major centre and the patient returning to a less urbanised environment after discharge was associated with reduced risk for psychiatric disorders. Tzeng et al. concluded that regular psychiatric follow-up is key for reducing the psychiatric burden of GBS [11].

Acute anxiety and delirium-like symptoms in GBS patients

GBS is an anxiogenic disease. Factors associated with anxiety in GBS include age, intensity of paralysis or disability, arm dysfunction, bulbar dysfunction, intensive care unit (ICU) admission, requirement for intubation or assisted ventilation, concerns over long-term paralysis, uncertainty over disease progression, and lack of clarity on future prognosis [21, 25]. In the acute phase of GBS, patients experience moderate-to-severe anxiety from the onset of muscle weakness to the initiation of treatment with plasmapheresis or IV Ig. Of the most severely disabled patients, 82% suffer with anxiety. In a study by Weiss et al. that included 49 GBS patients admitted to ICU, anxiety was the most common psychiatric symptom during the acute phase of the disease [20]. Depression, psychosis, emotional disturbances, hopelessness, and demoralisation were also observed. Another study by Cochen et al. that included 139 GBS patients admitted to ICU, reported that 19% of patients experienced vivid dreams, 30% experienced illusions, 60% experienced hallucinations, and 70% experienced delusions [36]. The presence of residual anxiety during the recovery phase has been associated with the presence of fatigue at discharge [28].

Residual fatigue in GBS patients

Fatigue is experienced by 38% to 86% of GBS patients [40]. It is characterised by persistent tiredness not overcome by rest or sleep, reducing the ability to sustain mental and physical effort [32]. Residual fatigue may persist for many years after recovery from physical disability. It is an important psychiatric symptom during the recovery phase that impacts on the patient’s ability to undertake exercise and rehabilitation, independent of the resolution or persistence of physical weakness [40]. The cause of residual fatigue is unknown. However, fatigue is exacerbated by sleep disturbance, which occurs in approximately 50% of patients and it is also associated with depression [27, 35].

Conflicting reports have failed to conclusively establish whether age and sex are risk factors for fatigue in GBS patients [28, 31, 33, 41]. However, if an association of fatigue with age or sex does exist, this may be explained by higher rates of endocrine dysfunction (i.e., hypothalamic-pituitary-adrenal (HPA) axis dysfunction) among female patients over 50 years of age [31]. Although a patient’s experience of fatigue may be influenced by their perception of the increase in effort required to carry out exercise, rehabilitation, or activities of daily living, prolonged activation of the HPA axis due to the intense stress experienced during the acute phase of GBS may contribute to neuropsychiatric fatigue through a state of physiological exhaustion [40].

The subjective nature of fatigue is also influenced by psychosocial factors, such as attitude, coping mechanisms, well-being, and social circumstances [40]. This subjective component makes an objective assessment of fatigue difficult due to its multidimensionality, confounding factors, and variable manifestation. Nevertheless, fatigue has an enormous impact on the patient’s quality of life, because it is influenced by, and contributes to physical disability and rehabilitation outcomes in GBS [33].

Depression in GBS patients

GBS patients have 4.8-fold increased risk for depression [11]. A study by Bahnasy et al. found significantly higher depression scores in GBS patients compared to controls (2.6-fold increased) [10]. The prevalence of depression among the most severely disabled GBS patients is high (67%) [20]. Although the severity of depression is not significantly correlated with rate of initial progression of physical effects of GBS, the severity of depression is positively correlated with the severity of anxiety, which positively correlates with the severity of muscle weakness [10]. Therefore, more severely disabled GBS patients may be more likely to develop psychological symptoms, with residual depression negatively affecting rehabilitation and quality of life [26, 42].

Close relatives of GBS patients

GBS affects not only the patient, but also close relatives (e.g., spouse) during the first few months of the illness. Bernsen et al. reported that relatives may find it difficult to manage feelings of uncertainty during the acute phase of GBS, and that 10% of relatives experienced financial problems as a result of extra travelling cost, childcare, or loss of income [37]. Seventy-two percent of close relatives experienced social dysfunction, psychological distress, and problems performing activities of daily living. At one and three months after GBS onset, relatives stress levels positively correlated with GBS severity, suggesting close relatives of the most severely disabled patients are more likely to develop psychosocial symptoms. Although psychiatric symptoms may improve over a 12-month period, relatives with residual symptoms (anxiety and depression) at one month were found to have worse psychiatric morbidity at six months [37].

A multidisciplinary team approach for the management of GBS

GBS causes significant psychological distress in patients who, until relatively recently, may not have had any significant health issues. Unpicking the relationship between physical and psychiatric symptoms to determine their impact on patients with a view to improving management is a complex task. Therefore, involvement of the multidisciplinary team at an early stage may be crucial for improving outcomes.

Fear and anxiety associated with the suddenness in onset and severity of physical disability during the acute phase of GBS may impact on the psychological status of the patient and, although most patients experience improvement in physical symptoms, many experience long-term severe residual anxiety, depression, and fatigue [10, 28]. The prevalence of anxiety and depression in GBS patients is higher in comparison to other patients admitted to ICU for other conditions [12]. One hypothesis to explain residual psychiatric sequelae is dysfunction of the HPA axis resulting from a prolonged stress response to the antecedent infection that triggered the autoimmune neuropathy [40]. However, an initial psychiatric assessment in GBS patients may provide valuable information for decisions regarding in-patient transfer to ICU. Severity of anxiety correlates with the requirement for assisted ventilation, and, as such, anxiety is predictive for in-patient ICU transfer and presents as a warning sign that may provide vital information to neurologists and intensivists [21]. Sharshar et al. found that the object of anxiety (i.e., uncertainty over prognosis), rather than intensity of anxiety, was significantly correlated with the requirement for assisted ventilation. This fact may make clinical assessment of anxiety simple in that quantification of anxiety severity may not be necessary.

During the rehabilitation phase, the use of selective serotonin reuptake inhibitors (SSRIs) and supportive psychotherapy may play a role in reducing the anxiety and depression experienced by GBS patients. In a case series by Brousseau et al., three GBS patients with anxiety and affective liability showed marked improvement after treatment with SSRIs and gabapentin [12]. Other pharmacological approaches for the management of the psychiatric effects of GBS that have been studied have included the use of the glutamate antagonist amantadine. Garssen et al. reported that amantadine was not different from placebo at reducing anxiety, depression, and fatigue in GBS patients [24]. A systematic review that assessed the quality of the evidence in Garssen et al. rated the level of evidence as poor, rendering the benefit of amantadine in GBS patients uncertain [24, 43].

Non-pharmacological approaches for the management of psychological symptoms include cognitive behavioural therapy (CBT) and occupational rehabilitation. These modalities may alleviate anxiety and depression caused by the social consequences of GBS, such as loss of employment, reduced ability to fulfil family responsibilities, and reduced social life [40]. CBT that promotes positive attitudes, coping mechanisms, and psychosocial support is also important for alleviating the effects of residual fatigue [44]. Therefore, psychoeducation and supportive therapy may reduce morbidity and increase quality of life by enhancing patients’ abilities at undertaking activities of daily living and participate in social life. Eisendraht et al. also suggested that education of family members is extremely important because it may recalibrate expectations, enabling relatives to adapt to the situation of caring for a GBS patient at home [23].

The early involvement of the physiotherapist is essential for psychiatric as well as physical recovery. Garssen et al. reported that physical exercise not only significantly reduces fatigue, but also relieves anxiety and depression [38]. The implementation of a tailor-made exercise programme reduces fatigue in GBS patients by 20% [4, 38]. Although physical fitness itself reduces fatigue levels and improves mental functioning, exercise also improves fatigue independent of the patient’s physical fitness level [29, 38]. According to de Vries et al., the mechanism underpinning exercise-dependent amelioration of residual fatigue from GBS involves the beneficial effects of exercise on the HPA axis that reduces the patient’s perception of fatigue [40]. Other factors that may reduce fatigue include peer support from other patients in a training group [29, 38].

Conflicting reports have suggested that not all GBS patients have favourable responses to exercise [45, 46]. A lack of improvement in residual fatigue may not be easy to rationalise, due to the absence of an underlying pathophysiological mechanism (such as nerve dysfunction, anaemia, hypothyroidism, diabetes, or chronic illness) [7, 30]. However, it is important to consider whether persistent fatigue may be due to a psychiatric mechanism (e.g., depression) that hampers recovery independent of exercise-induced improvements in muscle strength [28].

A recent study by Bahnasy et al. reported that sleep quality was significantly reduced in 20 GBS patients compared to healthy controls [10]. Severity of physical disability and initial anxiety were positively correlated with waking after sleep onset. Sleep disorders in GBS patients due to diaphragmatic paresis may also contribute to disrupted sleep patterns. Although it may be possible that sleep disturbance should contribute to development of residual fatigue, this has not been demonstrated conclusively in GBS patients [28]. Nevertheless, Bahnasy et al. suggested that GBS patients may benefit from treatment of sleep disturbances, either pharmacologically or with continuous positive airway pressure ventilation [10]. To promote long-term psychosocial independence, patients need to be aware of the importance of recognising and seeking treatment for the symptoms of fatigue, because the combined effects of acute physical fatigue and sleep disturbance may contribute to residual mental fatigue many years after rehabilitation.

## Conclusions

Psychological distress, which most often manifests as co-morbid anxiety, sleep disturbance and depression, may develop during, or even before, the onset of physical disability in GBS. The psychiatric symptoms of GBS should be identified early and recognised as representing more than mere ‘ICU delirium’. Management of GBS should be optimised to account for the psychiatric effects on the patient and close relatives. This may require a psychiatric assessment in the early stages of the disease as well as a multidisciplinary team approach to long-term management. As with any psychiatric illness, the clinician should pay close attention to a history of any other psychiatric conditions as well as other psychosocial factors that may increase risk, such as relationships, living conditions, social isolation/support, and family history. To support patients and families in the hospital setting and in the community, psychoeducation can be offered regarding self-care as well as services that provide information about mental health, including charities such as Mind (mind.org.uk) and newly-developed mental health apps (for a list of apps that have been assessed against UK NHS standards see: https://www.nhs.uk/apps-library/category/mental-health/). In addition to psychoeducation, low-intensity psychosocial interventions such as individual or group-based cognitive behavioural therapy (CBT), family therapy or counselling can also be considered. Although the evidence for complimentary therapies is mostly anecdotal, there may be no harm in discussing a range of alternative options if this will enable the patient to engage with therapy, such as acupuncture, yoga, biofeedback, relaxation, etc.

The detection and management of psychiatric symptoms during the acute phase of GBS may help to mitigate the contribution of these factors to the development of long-term physical residua. The ‘social skills’ of healthcare professionals play a critical role in preparing patients and close relatives for necessary adjustments at home. Dramatic improvements in long-term rehabilitation may be achieved through greater education and awareness of the psychological impact of GBS as well as adequate support and follow-up beyond the first year of recovery. Interestingly, two case studies identified patients in whom prior psychological symptoms preceded the development of muscle weakness and areflexia in GBS, suggesting that the mechanism of onset and the relationship between physical and psychiatric symptoms may be complex. Further research is required to understand the relationship between the onset of physical and psychiatric symptoms as well as the prognostic value and treatment of psychiatric sequelae of GBS.
